# Synergistic induction of apoptosis by salinomycin and gefitinib through lysosomal and mitochondrial dependent pathway overcomes gefitinib resistance in colorectal cancer

**DOI:** 10.18632/oncotarget.5628

**Published:** 2015-10-07

**Authors:** Zheng-Zhi Zou, Pei-Pei Nie, Ya-Wei Li, Ben-Xin Hou, Rui-Li, Xin-Peng Shi, Zhao-Kui Ma, Bao-Wei Han, Xiao-Yong Luo

**Affiliations:** ^1^ MOE Key Laboratory of Laser Life Science and Institute of Laser Life Science, College of Biophotonics, South China Normal University, Guangzhou, China; ^2^ Department of Oncology, The Affiliated Luoyang Central Hospital of Zhengzhou University, Luoyang, China; ^3^ Joint Laboratory of Laser Oncology with Cancer Center of Sun Yat-sen University, South China Normal University, Guangzhou, China; ^4^ KingMed Diagnostics and KingMed School of Laboratory Medicine, Guangzhou Medical University, Guangzhou, China; ^5^ Surgical department, The Affiliated Luoyang Central Hospital of Zhengzhou University, Luoyang, China; ^6^ Department of General Surgery, Hainan Province Nongken Sanya Hospital, Sanya, China

**Keywords:** salinomycin, gefitinib, synergistic, apoptosis, resistance, colorectal cancer

## Abstract

Here, we showed the antibiotic salinomycin (SAL) combined with GEF exerted synergistic cytotoxicity effects in colorectal cancer cells irrespective of their EGFR and KRAS status, with a relatively low toxicity to normal cells. Additionally, combination of the two drugs overcame Ras-induced resistance and the acquired resistance to GEF. Further, we identified a new potential mechanism of this cooperative interaction by showing that GEF and SAL acted together to enhance production of reactive oxygen species (ROS), loss of mitochondrial membrane potential (MMP) and lysosomal membrane potential (LMP). And the ROS contributed the loss of MMP and LMP. We also found that GEF and SAL acted in concert to induce apoptosis via a mitochondrial-lysosomal cross-talk and caspase-independent pathway triggered by cathepsin B and D. Lastly, SAL in combination with GEF sensitized GEF-resistant cells to GEF in a nude mouse xenograft model. This novel combination treatment might provide a potential clinical application to overcome GEF resistance in colorectal cancer.

## INTRODUCTION

Colorectal cancer is one of the leading causes of tumor-related death worldwide. Despite significant progress has been made in the treatment of patients with colorectal cancer in recent years, there is a constant demand for new therapies. Novel therapeutic strategies for colorectal cancer have been focused on developing targeted molecular therapies [1]. The epidermal growth factor receptor (EGFR) is a pivotal regulator of cell proliferation and progression, and widely expressed in several human cancer types (DEL) [2]. The EGFR is also frequently high expressed in human colorectal tumors relative to normal intestinal tissue, and this is dramatically associated with increased metastatic potential and poor prognosis of patients [3]. EGFR-targeted therapeutics has indicated clinical success in the treatment of several types of cancers, including colorectal cancer [4]. Gefitinib (GEF, brand name Iressa), an orally active EGFR inhibitor, has been approved for several types of tumor including colorectal cancer [5, 6]. However, the clinical efficacy of GEF introduced in the clinical practice for the therapy of colorectal cancers is limited to some patients showing either intrinsic or acquired resistance to GEF [7]. The recent progresses in the knowledge of the molecular mechanisms of GEF resistance have identified that the altered expression and activity of Ras and Raf, the down-stream signaling molecules of EGFR, exert a critical role in the resistance of cancer cells to GEF [8]. Moreover, the presence of activating mutations of KRAS, NRAS, and BRAF genes, as well as amplification of MET and HER2 genes in these colorectal cancers are reliable predictors of tumor resistance to GEF therapy [9, 10]. Combinations of GEF and the agents targeting other receptors or downstream effectors can overcome resistance to GEF has been shown in colorectal cancer cell lines [11]. However, the clinical studies that evaluated the combinations of GEF and the agents targeting other molecules have not demonstrated improvements in response or disease control rates [11].

The antibiotic salinomycin (SAL), a potassium ionophore, has been used as a veterinary drug for many years worldwide. Recent studies indicate that SAL exhibits a wide range of anticancer activities, including inhibition of proliferation, induction of autophagy, cell death and apoptosis in cancer cells [12]. Notably, the compound can effectively target tumor stem cells in several types of cancer, including leukemia, breast cancer and colorectal cancer, with a relatively low toxicity to normal cells [13]. More recently, SAL has been used to prevent the growth of chemoresistant cancer cells by inhibiting P-glycoprotein (P-gp), an efflux pump inhibitor [14]. In addition, several studies demonstrate SAL enhances the cytotoxic effects of several chemotherapeutic drugs including adriamycin and etoposide [15]. Based on above studies, we decided to determine if SAL can enhance the effect of GEF and overcome GEF resistance in colorectal cancer cells.

In the present study, we showed that the combination of GEF and SAL was synergistic at decreasing cell viability, colony formation ability and inducing cell apoptosis in colorectal cancer cell lines irrespective of their EGFR and KRAS status. Additionally, we identified a new potential mechanism of this cooperative interaction by showing that GEF and SAL acted together to enhance production of reactive oxygen species (ROS), loss of mitochondrial membrane potential (MMP) and lysosomal membrane potential (LMP). We also found that GEF and SAL treatment induced loss of LMP and MMP involved in ROS production. Moreover, GEF and SAL acted in concert to induce apoptosis via a mitochondrial-lysosomal cross-talk. Lastly, we showed that SAL in combination with GEF sensitized GEF-resistant cells to GEF in xenograft tumor models. Taken together, these accumulating data might guide development of new colon cancer therapies.

## RESULTS

### Synergistic antineoplastic effects induced by gefitinib and salinomycin overcome gefitinib resistance in colorectal cancer cells

**Figure 1:**
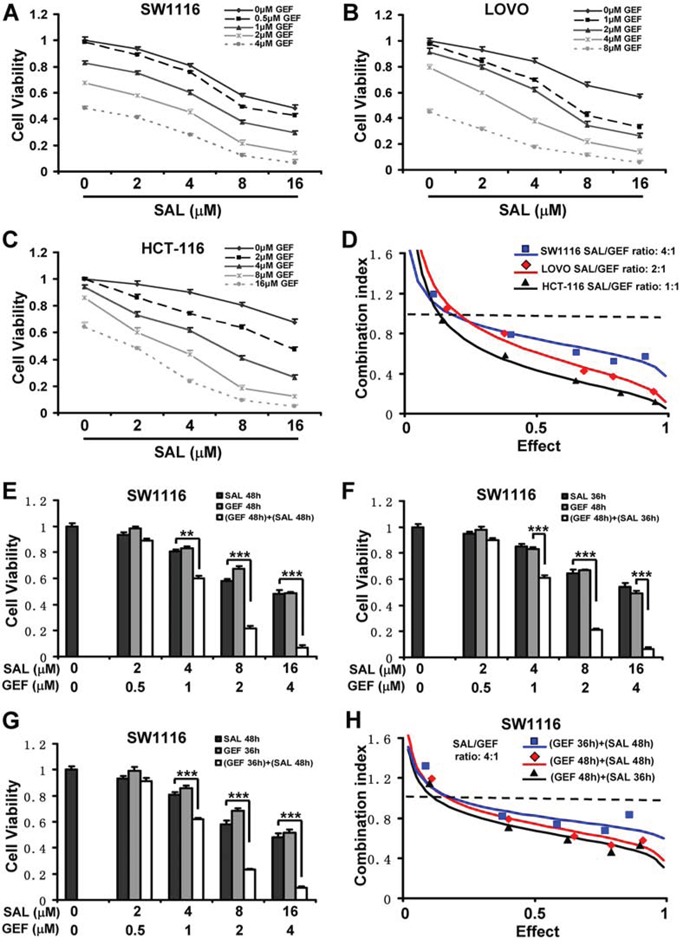
Synergistic antineoplastic effects induced by gefitinib and salinomycin overcome gefitinib resistance in colorectal cancer cells **(A-C)** Cell viability was assessed by CCK8 assay. Mean ± S.D. of three independent experiments performed in triplicate are shown. **D**. The CI values for gefitinib (GEF) and salinomycin (SAL) were calculated according to the Chou-Talalay's method at the 48 h time point, with the biological response being expressed as the fraction of affected (Fa) cells. Rectangle, diamond and triangle symbol designated the CI value for each Fa in SW1116, LOVO and HCT-116 respectively. The data are representative of three independent experiments. **E**. SW1116 cells were treated for 48 h with indicative dose of GEF and SAL. **F**. SW1116 cells were pretreated for 12 h with indicative dose of GEF and subsequentely with indicative dose of SAL for additional 36 h. **G**. SW1116 cells were pretreated for 12 h with indicative dose of SAL and subsequentely with indicative dose of GEF for additional 36 h. And then cells were assessed for viability by CCK8 assay. Mean ± S.D. of three independent experiments performed in triplicate are shown. **P < 0.01, ***P < 0.001. **H**. SW1116 cells were treated and processed as in E-G. The CI values for GEF and SAL were calculated according to the Chou-Talalay's method at the 48 h time point, with the biological response being expressed as the Fa cells. Rectangle, diamond and triangle symbol designated the CI value for each Fa in SW1116 cells with three different sequences of GEF and SAL. The data are representative of three independent experiments.

To determine the effects of gefitinib (GEF) and salinomycin (SAL) in combination on human colorectal cancer cell viability, SW1116, LOVO, HCT-116, SW480 and HT-29 cells with different EGFR status were exposed to increasing concentrations of GEF and SAL as single agent for 48 h respectively, and effects on cell viability were assessed by CCK8 assay. As shown in Table 1 and Figure 1, the IC_50_ doses for GEF ranged between 3 and 32 μM, and the IC_50_ doses for SAL ranged between 12 and 26 μM. SW1116 and HT-29 cells were relatively more sensitive to GEF; SW480 and LOVO cells showed a moderate sensitivity, conversely, HCT-116 cells were relatively more resistant to GEF. Additionally, by colony formation assay, the IC_50_ doses for GEF ranged between 1.5 and 13 μM, and the IC_50_ doses for SAL ranged between 10 and 29 μM in the five colorectal cancer cell lines (Table 1). Similarly, we found SW1116 and HT-29 cells were relatively more sensitive to GEF, whereas HCT-116 cells were relatively more resistant to GEF in colony formation assay.

**Table 1:** Effects of GEF and SAL as single agents in colorectal cancer and epithelial cell lines

Cell line	EGFR mutation	KRAS mutation (Codon 12WT = GGC)	KRAS mutation (Codon 13WT = GGC)	CCK8 analysis		Colony formation	
				GEF IC_50_ (μM)	SAL IC_50_ (μM)	GEF IC_50_ (μM)	SAL IC_50_ (μM)
SW1116	WT	WT	WT	4.1	12.5	2.6	10.9
LOVO	WT	WT	GAC	6.9	18.7	5.4	22.3
HCT-116	WT	WT	GAC	31.7	25.5	12.9	28.4
SW480	WT	GTT	WT	6.2	21.3	3.8	16.5
HT-29	WT	WT	WT	3.5	15.8	1.9	12.6
NCM460	WT	WT	WT	12.8	17.7	6.9	10.6

Cell growth inhibitory effects of salinomycin (SAL) and gefitinib (GEF) given as single agents were evaluated by CCK8 analysis and colony formation assay. IC_50_ values for each drug were calculated by performing dose–response experiments. For CCK8 analysis, cells were treated for 48 h with SAL (0.5–100 μM) and GEF (0.1–50 μM). For colony formation assay, cells were treated for 24 h with SAL (0.5–100 μM) and GEF (0.1–50 μM), after media removal, cells were cultured with fresh medium for additional 15 days. Experiments were performed in triplicate.

To evaluate the potential synergistic effects of GEF and SAL against colorectal cancer cell, all five cells lines were treated with increasing doses of GEF and SAL alone or in combination, and effects on cell viability were assessed by CCK8 assay and colony formation assay. As shown in Figure 1A, B and C, combination of GEF and SAL had a stronger inhibitory effect on the cell viability of colorectal cancer cells than either drug alone. These data implied that the GEF and SAL might synergize to prevent cell viability in colon cancer cells. To confirm this synergism, all five colorectal cancer cell lines were treated with a combination of the two compounds in a constant ratio to one another, and combination index (CI) was calculated using Calcusyn software following Chou and Talalay's method. As shown in Figure 1C and Table 2, significant synergies between the two agents (CI < 1) were found in the five colorectal cancer cells. Notably, we also treated the NCM460 cells, a normal human colorectal mucosal epithelial cell line, with SAL and GEF in combination, and found the two drugs in combination displayed antagonistic or minimal synergistic effects in the normal immortalised cell lines (Table 1 and 2).

**Table 2:** Effects of GEF and SAL in combination in colorectal cancer and epithelial cell lines

Cell line	SAL/GEF	CCK8 analysis				Colony formation			
		CI at Fa_30_	CI at Fa_50_	CI at Fa_75_	CI at Fa_90_	CI at Fa_30_	CI at Fa_50_	CI at Fa_75_	CI at Fa_90_
SW1116	4:1	0.86	0.74	0.62	0.52	0.42	0.35	0.27	0.19
LOVO	2:1	0.82	0.61	0.41	0.28	0.36	0.27	0.22	0.17
HCT-116	1:1	0.63	0.43	0.26	0.16	0.28	0.21	0.16	0.10
SW480	4:1	0.89	0.76	0.63	0.49	0.45	0.33	0.28	0.21
HT-29	4:1	0.79	0.71	0.58	0.35	0.40	0.32	0.25	0.19
NCM460	2:1	1.82	1.51	1.28	1.17	1.65	1.42	1.25	0.95

Synergistic growth inhibitory effects of the combined treatment with SAL and GEF were evaluated by CCK8 analysis and colony formation assay. Experiments were performed in triplicate. CI values were calculated according to the Chou-Talaly mathematical model for drug interactions using the Calcusyn software for different fractions affected (Fa). CI is a quantitative measure of the degree of interaction between different drugs. CI = 1, additivity; CI > 1, antagonism; CI < 1, synergism.

Next we asked whether SAL might enhance the cytotoxicity of other EGFR inhibitors, SW1116 cells were treated with SAL combined with four other EGFR inhibitors erlotinib (ERL), AEE-788 (AEE), afatinib (AFA), dacomitinib (DAC) respectively. Notably, although SAL enhanced the toxic and apoptotic effects of ERL, AEE, AFA and DAC in SW1116 cells, the effects were very weak (Supplementary Figure 1A, C, E and F). Moreover, we showed that the phosphorylated EGFR were significantly decreased by these EGFR inhibitors (Supplementary Figure 1G and H). However, by calculating the CI, we found ERL, AEE, AFA and DAC combined with SAL displayed antagonistic effects (CI > 1, Supplementary Figure 1B and D).

As AKT signaling pathway stimulates cancer cell growth and inhibits cell apoptosis, we determined the effects of GEF and SAL on the activation of AKT. As shown in Supplementary Figure 2A and B, We observed GEF and SAL obviously reduced the phosphorylation of AKT in SW1116 and HCT-116 cells.

Some previous studies found that the synergistic effect induced by GEF combined with chemotherapy drugs was dependent on the sequence of administration of these agents [16]. Therefore, we evaluated the cytotoxic effects of different treatment schedules of GEF and SAL in SW1116 and HCT-116 cells. Three schedules were performed in this study: first, GEF followed by SAL; second, concurrent administration; third, SAL followed by GEF. As shown in Figure 1G-H and Supplementary Figure 3A-D, all three sequential administrations of the two agents exerted clear synergistic effects (CI < 1) in SW1116 and HCT-116 cell lines, suggesting the synergistic effect induced by GEF combined with SAL was independent of the sequence of administration.

### Combination of salinomycin and gefitinib overcomes gefitinib resistance in Ras-overexpressing and acquired gefitinib-resistant colorectal cancer cells

**Figure 2:**
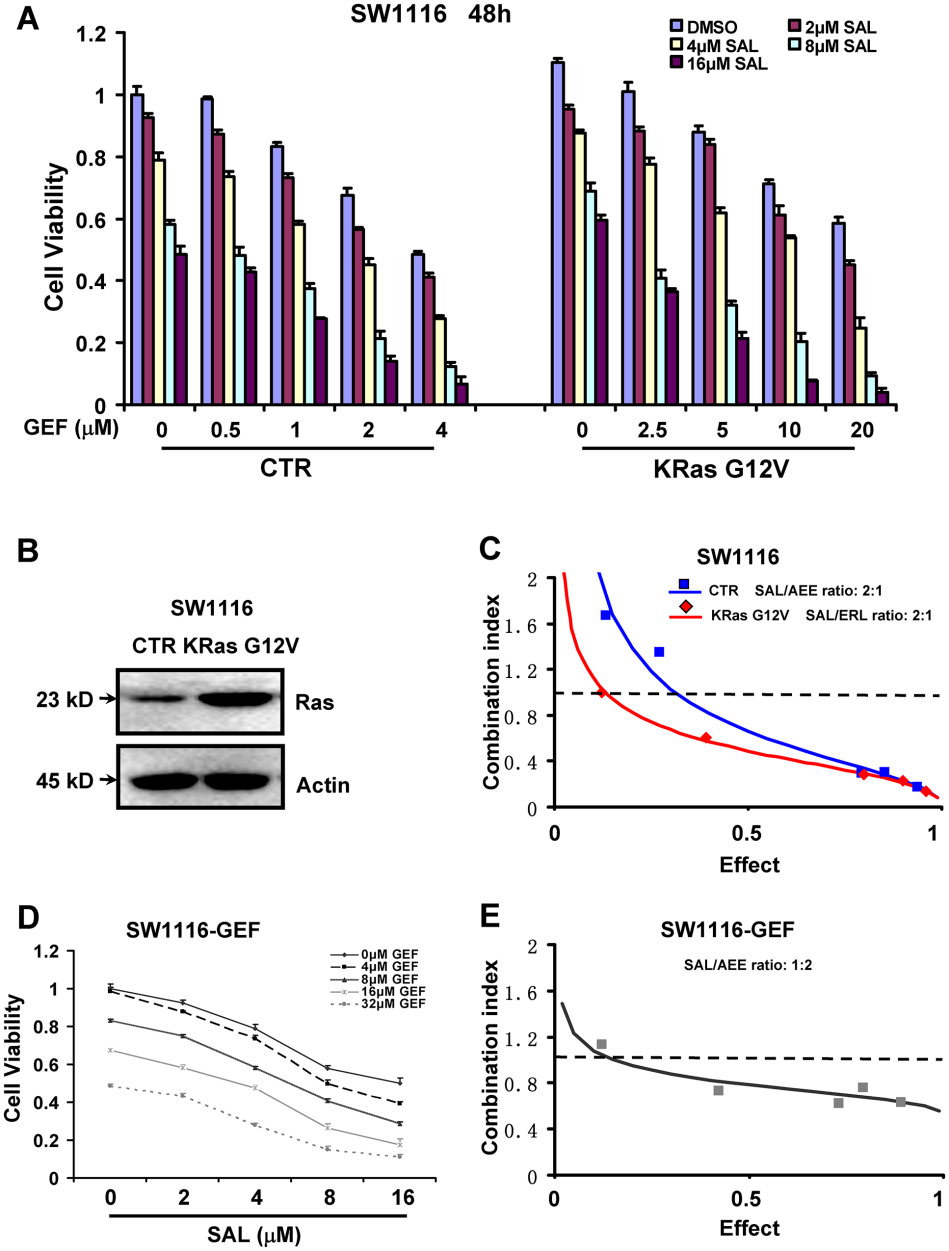
Combination of salinomycin and gefitinib overcomes gefitinib resistance in Ras-overexpressing and acquired gefitinib-resistant colorectal cancer cells **A**. SW1116 cells were transfected with control (Ctr) and KRas G12V vector and then treated with gefitinib (GEF) and salinomycin (SAL), alone or in combination, and then assessed for viability by CCK8 assay. **B**. Effects of KRas overexpression were analysed by Western blot analysis. **C**. The combination index (CI) values for GEF and SAL were calculated according to the Chou-Talalay's method, with the biological response being expressed as the fraction of affected (Fa) cells. Rectangle and diamond symbol designated the CI value for each Fa in Ctr and KRas G12V vector transfected SW1116 cells respectively. **D**. Cell viability was assessed by CCK8 assay in the acquired GEF-resistant SW1116-GEF cells. Mean ± S.D. of three independent experiments performed in triplicate are shown. **E**. The CI values for GEF and SAL were calculated according to the Chou-Talalay's method at the 48 h time point, with the biological response being expressed as the fraction of affected (Fa) cells. The data are representative of three independent experiments.

Mutational activation of K-Ras is involved in primary resistance of GEF in colorectal cancer [17]. To confirm whether combination of SAL and GEF can overcome activation of K-Ras induced GEF resistance, SW1116 cell line, which was relatively more sensitive to GEF, was transiently transfected with constitutional activating mutational K-Ras (G12V) plasmids. And then, overexpressing mutational K-Ras (G12V) and control SW1116 cells cells were treated with increasing concentration of GEF, and SAL alone or in combination. As shown in Figure 2A, transfection of mutant K-Ras (G12V) plasmids could remarkably inhibit the GEF cytotoxicity with the IC_50_ value of GEF increased to 22 μM. In addition, the transfection efficacy of K-Ras (G12V) plasmid was determined by Western blotting (Figure 2B). To confirm whether combination of SAL and GEF displayed synergism in SW1116 cells overexpressing K-Ras (G12V), CI was calculated using Calcusyn software following Chou and Talalay's method. As shown in Figure 2C, significant synergies between the two agents (CI < 1) were found in the cells.

Moreover, we modeled the development of acquired GEF resistance in patients by treating SW1116 cells with increasing concentrations of GEF for 6 months to select GEF-resistant cells (SW1116-GEF) (Supplementary Figure 4A). As shown in Figure 2D, the SW1116-GEF cells were significantly more resistant (8.2 folds) to GEF treatment relative to the parental cells *in vitro*. Moreover, we found SW1116-GEF cells exerted higher invasive potency and expressed higher levels of p-AKT (Supplementary Figure 4B and C). Next we examined whether SAL might enhance the cytotoxicity of GEF in SW1116-GEF cells. Figure 2D and E indicated that GEF combined with SAL exerted significant synergistic cytotoxic activity (CI < 1) against SW1116-GEF cells. These results suggested that combination of SAL and GEF could overcome gefitinib resistance in acquired gefitinib-resistant colorectal cancer cells.

### Synergistic induction of apoptosis in colorectal cancer cells by gefitinib and salinomycin

**Figure 3:**
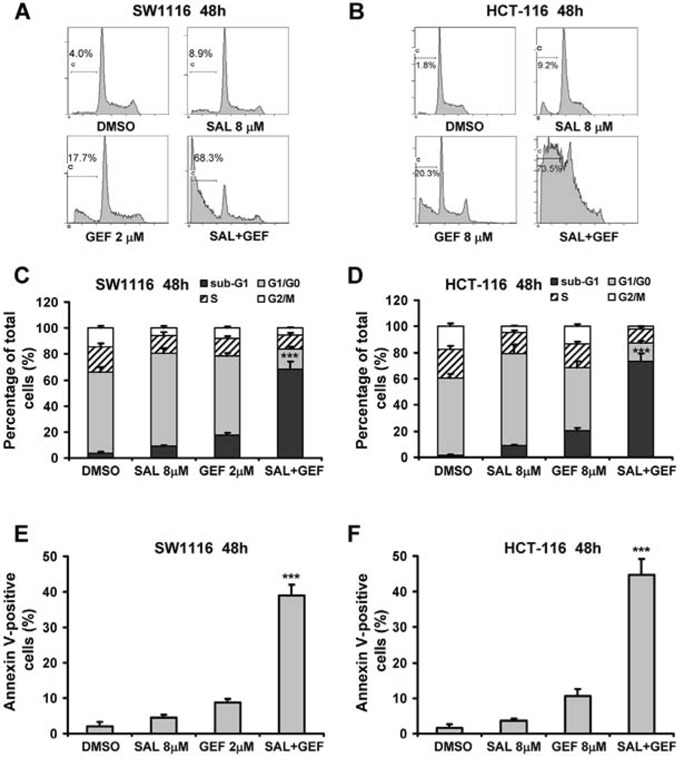
Synergistic induction of apoptosis in colorectal cancer cells by gefitinib and salinomycin **A**. and **B**. SW1116 and HCT-116 cells were treated with indicated concentrations of gefitinib (GEF) and salinomycin (SAL) alone and in combination for 48 h. After the cells were stained with propidium iodide, and the DNA content was measured by flow cytometry. Results shown are representative of three independent experiments. **C**. and **D**. The proportion of nuclei at each phase of cell cycle was obtained using FlowJo V7 DNA analysis software. Columns, mean of three determinations; bars, S.D. **E**. and **F**. Cells were treated with indicated concentrations of GEF and SAL for 48 h. Cell apoptosis was assessed by Annexin V-FITC/PI staining assay by flow cytometry. Columns, mean of three determinations; bars, S.D., ***P < 0.001, compared with DMSO-treated cells.

To determine if the cytotoxic effects of GEF plus SAL were due to induction of apoptosis, SW1116 and HCT-116 cells were treated with different concentration of GEF combined with SAL for 48 h, and then cell apoptosis was determined by detecting sub-G_1_ population with propidium iodide (PI) staining and flow cytometry analyses. As shown in Figure 3A-D, the sub-G_1_ population percentages triggered by the treatments with the two agents combination were greater than those induced by the compounds individually. Additionally, the synergistic apoptotic effect of the drug combination was further confirmed by Annexin V-FITC and PI staining and flow cytometry analysis. Figure 3E-F indicated that colorectal cancer cells treated with two compounds in combination showed significant increase in the proportion of Annexin V-positive cells.

### Gefitinib and salinomycin in combination treatment induces cell apoptosis involved in ROS production

Previous studies have shown that SAL induces colon and breast cancer cell apoptosis by increasing intracellular ROS levels [12]. To examine whether SAL combined with GEF was able to cause cell apoptosis via increased ROS production in colorectal cancer cells, SW1116 and HCT-116 cells were treated for 48 h with SAL and GEF alone or in combination, and then the levels of ROS were detected by DCFH-DA staining and flow cytometric assays. As shown in Figure 3A-D, GEF markedly enhanced the levels of ROS induced by SAL in both colorectal cancer cells. However, GEF alone failed to stimulate the production of ROS in the two cell lines. Additionally, we detected the levels of ROS in SW1116 cells treated with 2 μM of GEF in combination with 8 μM SAL for different time (2–32 h). As shown in Figure 3E-F, the levels of ROS gradually increased in a time-dependent manner during 0–24 h. The highest concentrations of ROS were found at the 24 h time points. Notably, other EGFR inhibitors AEE and ERL were completely incapable of increasing the ROS levels induced by SAL (Supplementary Figure 5).

**Figure 4:**
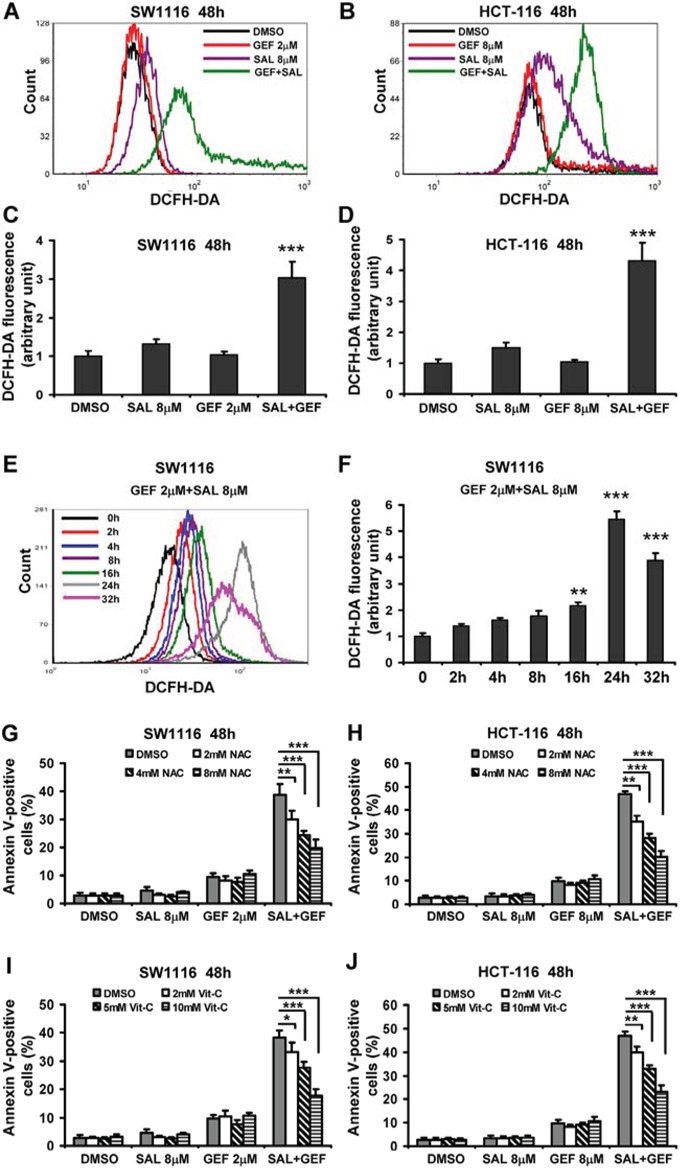
Gefitinib and salinomycin in combination treatment induces cell apoptosis involved in ROS production in colorectal cancer cells **A**. and **B**. The levels of ROS were measured with DCFH-DA staining by flow cytometric analyses at 48 h after gefitinib (GEF) and salinomycin (SAL) treatment in SW1116 and HCT-116 cells. **C**. and **D**. The levels of ROS were presented as fold change compared to the levels in control cells. Columns, mean of three determinations; bars, S.D. Results shown are representative of three independent experiments. **E**. The levels of ROS were measured with DCFH-DA staining by flow cytometric analyses at indicated time point after treatment of GEF and SAL in combination in SW1116 cells. **F**. The levels of ROS were presented as fold change compared to the levels in control cells. Columns, mean of three determinations; bars, S.D. Results shown are representative of three independent experiments. **(G-J)** SW1116 and HCT-116 cells were pretreated with different concentration of N-acetyl-L-cysteine (NAC) and vitamin C (Vit-C, dehydroascorbic acid) for 30 min before GEF and SAL treatment. And then cells were treated with indicated concentrations of GEF and SAL alone and in combination for additional 48 h. Cells apoptosis was measured using an Annexin V-FITC/PI staining assay. *P < 0.05 vs. control; **P < 0.01 vs. control; ***P < 0.001 vs. control.

To further investigate whether enhanced production of ROS correlated with increase of cell apoptosis, we pretreated colorectal cancer cells with ROS scavengers N-acetyl-L-cysteine (NAC) and vitamin C (Vit-C) prior to SAL plus GEF treatment to determine whether inhibition of ROS production could block cell apoptosis induced by the two drugs. As shown in Figure 4G-J, pretreatment with NAC and Vit-C significantly decreased cells apoptosis. In addition, the inductions of ROS by GEF plus SAL were completely blocked by pretreatment with 8 mM NAC or 10 mM Vit-c in SW1116 and HCT-116 cells (Supplementary Figure 6).

These results suggested that SAL enhanced the cytotoxic effects of GEF might be associated with ROS-increased cell death. Since NAC and Vit-C could completely abolish ROS burst induced by the two agents, while SAL plus GEF induced apoptosis was only partially prevented by NAC or Vit-C, these results hinted some other apoptotic molecules besides ROS were involved in the cell apoptosis evoked by SAL and GEF in combination.

### Gefitinib and salinomycin cooperate to trigger loss of lysosomal membrane potential and mitochondrial membrane potential

**Figure 5:**
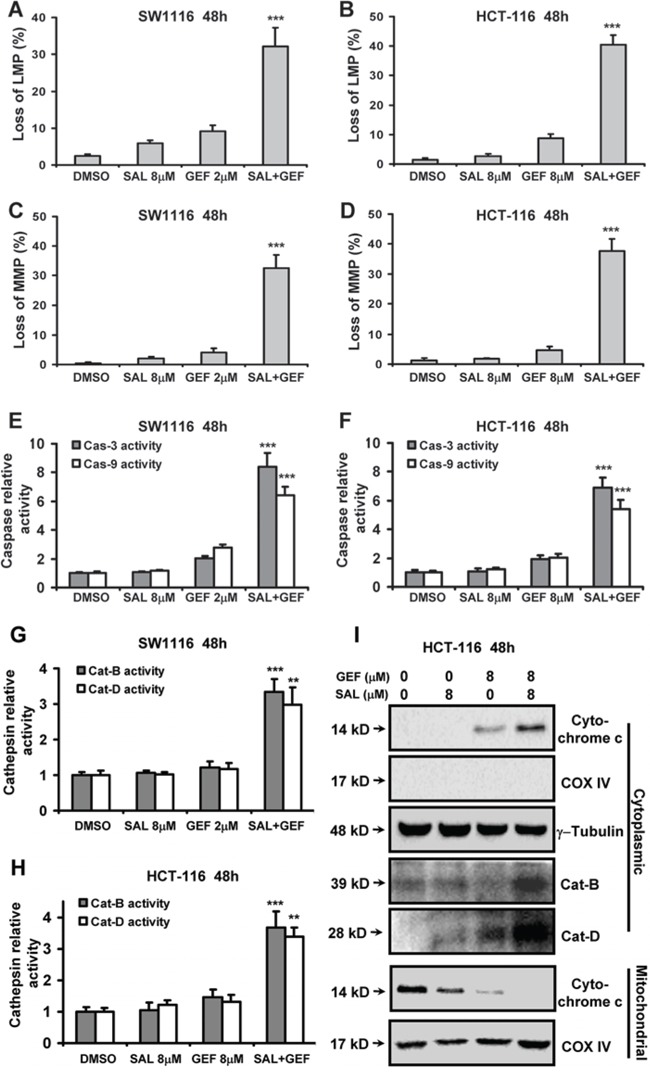
Gefitinib and Salinomycin cooperate to trigger loss of lysosomal membrane potential and mitochondrial membrane potential **A**. and **B**. The levels of LMP were measured with Acridine Orange (AO) staining by flow cytometric analyses at 48 h after GEF and SAL treatment. The percentage of cells with loss of LMP was shown. **C**. and **D**. The levels of MMP were measured with JC-1 staining by flow cytometric analyses at 48 h after GEF and SAL treatment. The percentage of cells with loss of MMP was shown. **E-H**. The caspase and cathepsin activities were quantified as described under Methods. This experiment was repeated thrice. Columns, mean; bars, S.D. *P < 0.05 vs. control; **P < 0.01 vs. control; ***P < 0.001 vs. control. **I**. SW1116 cells were treated for 48 h with the indicated concentrations of GEF and SAL either alone or in combination, fractionated into cytosol and mitochondria, and analyzed for the distribution of cytochrome c and cathepsin B (Cat-B) and cathepsin D (Cat-D), by Western blot analysis. The fractionation quality was verified by the distribution of specific subcellular markers: COX IV for mitochondria and actin for cytosol. This experiment was repeated thrice.

Since the generation of ROS can cause lysosomal and mitochondrial dysfunction [18], we next monitored the lysosomal membrane potential (LMP) and mitochondrial membrane potential (MMP) by acridine orange (AO) and JC-1 staining respectively. SW1116 and HCT-116 cells were treated for 48 h with the indicated concentrations of GEF and SAL either alone or in combination. And then, the LMP and MMP were measured with AO and JC-1 staining by flow cytometric analyses. We found treatment with GEF and SAL in combination markedly increased loss of LMP and MMP (Figure 5A-D). Notably, we found AEE or ERL combined with SAL exerted minimal effects on the loss of MMP, whereas the drugs combinations were incapable of inducing the loss of LMP (Supplementary Figure 7).

Mitochondrial dysfunction is known to play a vital role in apoptosis by releasing cytochrome c into the cytoplasm to activate initiator caspase-9, thereby sequentially activate caspase-3, an executioner caspase. To further determine the loss of LMP and MMP induced by GEF combined with SAL, the activities of caspase-3 and -9 were measured by fluorogenic substrate cleavage. As shown in Figure 5E and 5F, colorectal cancer cells treated with two drugs in combination showed apparently upregulation in caspase-3 and -9 activities. Additionally, SAL combined with GEF significantly increased the amounts of cytochrome c in cytoplasm, whereas the mitochondrial cytochrome c levels were sharply attenuated by the two agents in combination (Figure 5I).

Since cell apoptosis involving the loss of lysosomal membrane potential has been described to involve the lysosomal proteases cathepsins [18], the activities of cathepsin B and D in cytoplasm were measured by fluorogenic substrate cleavage. As shown in Figure 5G and 5H, SW1116 and HCT-116 cells treated with SAL plus GEF in combination showed apparently upregulation in cathepsin B and D activities. Moreover, by Western blot analysis of cytosolic and mitochondrial fractions, we found that SAL plus GEF triggered redistribution of cathepsin B and D into the cytoplasm (Figure 5I), suggesting lysosomal leakage induced by the two drugs in combination.

In addition, the pan caspase inhibitor z-VAD-fmk (20 μM) was used to pretreat SW1116 or HCT-116 cells before treatment of GEF plus SAL. As shown in Supplementary Figure 8, z-VAD-fmk could completely block the activities of caspase-3 triggered by the two agents in combination, whereas cells apoptosis were not completely suppressed by z-VAD-fmk. These results indicated that GEF combined with SAL induced apoptosis involved caspase-dependent and caspase-independent pathways in colorectal cancer cells. Moreover, SW1116 and HCT-116 cells were pretreated with Necrostatin 1 (Nec1) and Necrostatin 5 (Nec5), inhibitors of necrotic cell death, and then treated with GEF plus SAL. We found that Nec1 and Nec5 did not attenuate cytotoxicity induced by GEF plus SAL in SW1116 and HCT-116 cells (Supplementary Figure 8E and F). These results implied SAL plus GEF induced colorectal cancer cell death was not involved in necrotic cell death.

### ROS generation contributes to loss of both lysosomal membrane potential and mitochondrial membrane potential induced by gefitinib and salinomycin in combination

**Figure 6:**
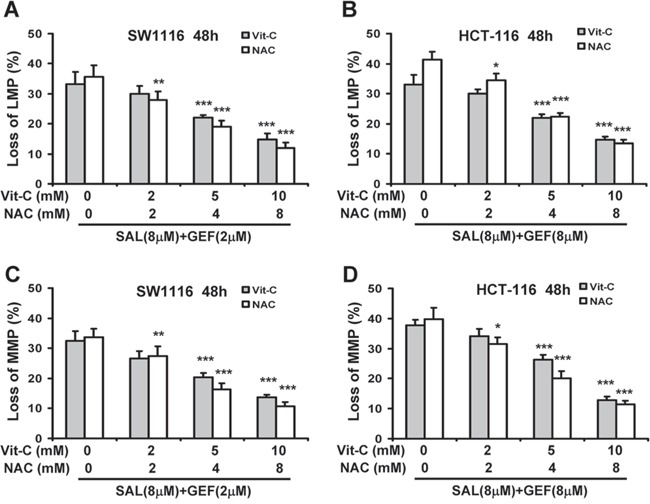
ROS generation contributes to loss of both lysosomal membrane potential and mitochondrial membrane potential induced by gefitinib and salinomycin in combination **(A-D)** SW1116 and HCT-116 cells were pretreated with different concentration of N-acetyl-L-cysteine (NAC) and vitamin C (Vit-C, dehydroascorbic acid) for 30 min before GEF and SAL treatment. And then cells were treated with indicated concentrations of GEF and SAL in combination for additional 48 h. Subsequently, the levels of LMP and MMP were measured with AO and JC-1 staining by flow cytometric analyses respectively columns, mean of three to four independent triplicate experiments; bars, S.D. *P < 0.05 vs. control; **P < 0.01 vs. control; ***P < 0.001 vs. control.

To better assess the significance of ROS generation in loss of both LMP and MMP induced by GEF and SAL in combination, SW1116 and HCT-116 cells were pretreated with increasing concentrations of ROS scavengers NAC and Vit-C, and then cells were treated with indicated concentrations of GEF and SAL in combination. As shown in Figure 6A-6D, both NAC and Vit-C significantly attenuated the loss of both LMP and MMP in dose-dependent manner in the two colorectal cancer cells treated with GEF and SAL in combination. These data indicated that ROS contributed to the loss of both LMP and MMP induced by the two compounds in combination.

### The loss of mitochondrial membrane potential induced by gefitinib and salinomycin in combination involved in cathepsin B and D

**Figure 7:**
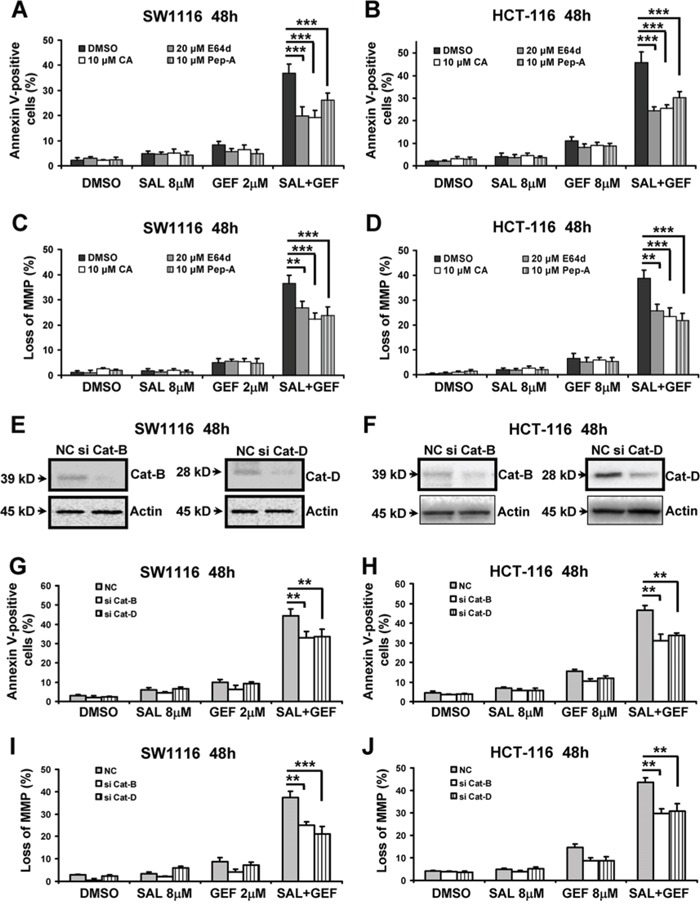
The loss of mitochondrial membrane potential induced by gefitinib and salinomycin in combination involved in cathepsin B and D in colon cancer cells **A–D** SW1116 and HCT-116 cells were pretreated with indicated concentration of E64d, CA-074-Me (CA) and pepstatin A (Pep-A) for 1 h were treated with indicated concentrations of GEF and SAL alone and in combination for additional 48 h. Cells apoptosis was measured using an Annexin V-FITC/PI staining assay. The levels of MMP were measured with JC-1 staining by flow cytometric analyses. columns, mean of three to four independent triplicate experiments; bars, S.D. **E-J** SW1116 and HCT-116 cells cells were transfected with 100 nM NC (negative control) siRNA, cathepsin B (Cat-B) siRNA and cathepsin D (Cat-D) siRNA respectively. Eight hours later, cells were treated with indicated concentrations of GEF and SAL alone and in combination for additional 48 h respectively. (E) and (F) The knockdown effects on Cat-B and Cat-D were confirmed by Western blot analysis. (G) and (H) Cell apoptosis was measured using an Annexin V-FITC/PI staining assay. (I) and (J) The levels of MMP were measured with JC-1 staining by flow cytometric analyses. columns, mean of three to four independent triplicate experiments; bars, S.D. *P < 0.05 vs. control; **P < 0.01 vs. control; ***P < 0.001 vs. control.

The data shown above indicated the combination treatment with GEF and SAL caused loss of LMP. Next, we investigated whether lysosomal dysfunction were involved in the synergistic induction of the loss of MMP by the two agents. Since lysosome-mediated apoptosis is associated with lysosomal cathepsin proteases released into the cytoplasm, we used several different inhibitors of cathepsins to pretreat colorectal cancer cells, and subsequently cells were incubated with GEF and SAL. As shown in Figure 7A and 7B, the addition of E64d (cathepsin B/L inhibitor), pepstatin A (Pep-A, inhibitor of cathepsin D/E) and CA-074-Me (CA, a selective irreversible inhibitor of cathepsin B) all evidently reduced cells apoptosis induced by GEF and SAL in combination. Moreover, we examed the effects of the three cathepsin inhibitors on the loss of MMP triggered by GEF combined with SAL. Figure 7C and 7D indicated that the loss of MMP was significantly attenuated by the three cathepsin inhibitors.

To further explore cathepsins contributed to cells apoptosis and loss of MMP induced by GEF and SAL, cathepsin B and D were depleted by using siRNAs. Figure 7E and 7F indicated that cathepsin B and D siRNAs effectively interfered with the expression of cathepsin B and D individually. Moreover, depletion of cathepsin B or D resulted in significant decrease of apoptosis induced by the two compounds in SW1116 and HCT-116 cells (Figure 7G and 7H). In addition, as shown in Figure 7I and 7J, both cathepsin B and D siRNAs significantly blocked the loss of MMP. The above experiments suggested that SAL and GEF cooperated to trigger LMP and cytoplasmic release of cathepsin B and D, which contributed to the cooperative loss of MMP and subsequently apoptosis.

In addition, the loss of LMP induced by SAL plus GEF in SW1116 cells were evidently prevented by the mitochondrial permeability transition (MPT) pore sealing agents cyclosporine and carnitine (Supplementary Figure 9). Together, this set of experiments suggested that the two drugs cooperated to trigger mitochondrial permeability, which contributed to the cooperative loss of LMP.

### Gefitinib and salinomycin synergistically overcome gefitinib resistance in xenograft tumor models

**Figure 8:**
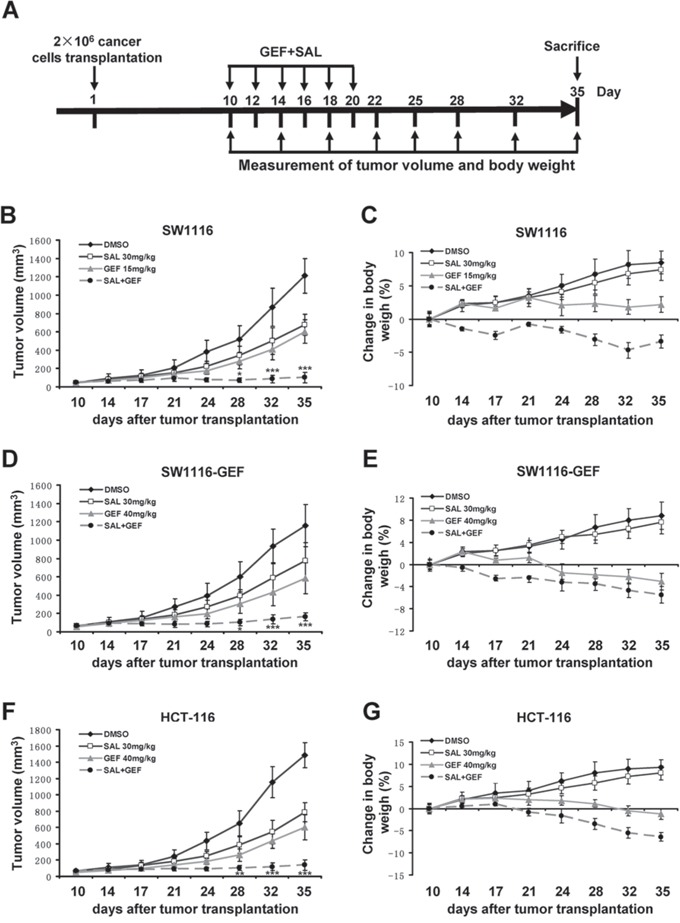
Gefitinib and salinomycin synergistically inhibit tumor growth in xenograft tumor models **A**. Treatment schedule. **B**, **D** and **F**. Combination of suboptimal concentrations of gefitinib (GEF) and salinomycin (SAL) displays significantly greater efficacy compared with either drug alone in xenograft tumor models. Nude mice bearing SW1116, SW1116-GEF and HCT-116 xenografts were administered with the indicated treatments. (For SW1116, GEF: 15 mg/kg; SAL: 30 mg/kg; for SW1116-GEF and HCT-116, GEF: 40 mg/kg; SAL: 30 mg/kg). Tumor volume was measured two times a week by using calipers (as indicated at each time point) for 25 days. Data are shown as mean ± S.D. (*n* = 6 per group). *P < 0.05; **P < 0.01; ***P < 0.001 vs. control. **C**, **E** and **G**. Average body weight changes were measured over the course of the study.

To test whether GEF combined SAL could be an effective strategy to overcome GEF resistance *in vivo*, we further evaluated the antitumor activity of SAL/GEF in xenografts established in nude mice implanted with SW1116 cells (relatively more sensitive to GEF), SW1116-GEF cells (acquired resistance to GEF) and HCT-116 cells (relatively more resistant to GEF). BALB/c-nude mice bearing xenograft tumors were administered SAL and GEF alone or combination by intraperitoneal injection once per two days for 10 days. Growth of tumors was measured twice weekly during the experimental period. The body weight of mice was recorded throughout the experiments. As shown in Figure 8B, 8D and 8F, while either SAL or GEF alone inhibited the tumors growth, their combination exerted a much stronger antitumor effects in both SW1116 and HCT-116 xenograft tumor models (*P* < 0.01). In addition, no significant increase in body weight loss was observed in the mice treated with the drugs (Figure 8C, 8E and 8G), suggesting the side effects of the two drugs were minimal *in vivo*.

## DISCUSSION

GEF has shown promising activity in a minority of colorectal cancer patients with high EGFR signaling activity [4]. However, the emerging clinical experience has regrettably revealed that GEF exerted very low effects in some colorectal cancer patients with *de novo* resistance or acquired resistance to the drug [19]. EGFR mainly activates the RAS pathway, promotes cell proliferation and facilitates cancer progression [20]. RAS, a small GTP binding protein, activates downstream RAF/MEK/ERK pathway and participates in many cellular processes including cell cycle and apoptosis [21]. The activating mutations in the RAS pathway result in desensitization of tumor cell to EGFR inhibition [21]. Moreover, clinical studies also have found that activating mutations in the *KRAS, RAF* and *MEK* proto-oncogenes enhance colorectal cancer resistance to GEF [22]. Accordingly, one current strategy to overcome colorectal cancer cell resistance to EGFR inhibitors is by inhibiting RAS pathway [23]. Additionally, in order to overcome resistance, EGFR inhibitors have been combined with small molecules targeting antiapoptotic factors, such as Bcl-2 [24]. Interestingly, in this study, we found the antibiotic SAL could overcome GEF resistance in *de novo* resistance colorectal cancer cell line HCT-116, acquired resistance cancer cell SW1116-GEF and RAS transient overexpressing induced GEF resistance cells. Notably, synergistic cytotoxicity of SAL and GEF was specific to colorectal cancer cells when compared with normal colorectal epithelial cells. This implied the combination of the two drugs might have a lower toxicity *in vivo* and be a novel therapeutic strategy against human colon cancer.

The novelty of this study resided in the demonstration that inductions of ROS, loss of LMP and MMP rather than inhibition of EGFR signal pathway were critical for the synergistic cytotoxicity of the drugs in combination. Since the combination of SAL and other two EGFR inhibitors AEE and ERL failed to induce the loss of LMP and ROS burst, these results suggested GEF enhanced the cytotoxicity of SAL by inhibiting other molecules not EGFR. Previous studies showed GEF inhibits transmembrane transporters of the ABC family, including the P-glycoprotein (P-gp), the multidrug resistance protein 1 (MRP1) and the breast cancer resistance protein (BCRP) to exert anticancer effects *in vivo* and *in vitro* [25].

SAL is a monocarboxylic polyether antibiotic used to inhibit coccidiosis in poultry [26]. As an anticancer drug, SAL induces cell apoptosis and prevents cell proliferation have been reported in breast, head and neck squamous cell, pancreatic and colorectal cancer [27–29]. More importantly, the cancer stem cells (CSCs) inhibiting activity of SAL has previously been demonstrated in a variety of tumors [30]. SAL has also been shown to overcome ABC transporter-enhanced multidrug resistance in leukemia and sarcoma stem cells [13, 14]. Moreover, SAL induces apoptosis in breast and colon cancer cell lines by promoting the formation of ROS [31]. Consistent with previous results, we also observed ROS generation in colorectal cancer cells after treatment with SAL. Furthermore, in this study, the production of ROS induced by SAL was sharply exaggerated by GEF. The ROS production stimulated the loss of LMP and MMP, and subsequent induction of apoptosis in colorectal cancer cells because ROS scavengers NAC and Vit-C, could markedly attenuate the Annexin-V positive cell populations.

Direct damage to the lysosomal and mitochondrial membrane by ROS during oxidative stress has been extensively reported [18, 32]. Moreover, indirect damage of the lysosomal membrane by ROS is mediated by the iron content of lysosomes. Hydrogen peroxides (H_2_O_2_), a ROS species, penetrate the lysosome and are converted into highly reactive hydroxyl radicals due to the intralysosomal accumulation of iron. These radicals are known to effectively damage cell membranes by lipid peroxidation [33]. Since the mitochondrial respiratory chain is the major source of ROS production [34], SAL combined GEF might target the mitochondrial respiratory chain to induce ROS production. The underlining mechanisms of ROS production triggered by the two drugs in combination need be further explored.

We observed that Cat-D and Cat-B were released from the lysosome to the cytosol in response to SAL plus GEF. Moreover, we showed inhibition of lysosomal cathepsin activities and depletion of Cat-B and -D using siRNAs significantly reduced loss of MMP and apoptosis. Previous studies reported that cathepsin-cleaved Bid promotes loss of MMP and apoptosis by inducing Bax-mediated release of cytochrome c from mitochondria [35]. In addition to Bid, antiapoptotic Bcl-2, Bcl-xL, Mcl-1 and XIAP proteins have been shown to be cleaved by cathepsins during induction of apoptosis of various malignant cells, including human colon carcinoma cells [36]. These results implied that the lysosomal damage induced by SAL combined with GEF contributed the mitochondrial permeability by releasing lysosomal Cat-B and -D into cytosol. Besides, inhibition of caspases by z-VAD-fmk only partially decreased GEF plus SAL induced apoptosis, clearly indicating that a caspase-independent apoptotic pathway was involved. Some studies have suggested caspase-independent cell death is promoted by lysosomal cathepsins in cytosol induced by chemotherapy drugs [37, 38]. Increased amounts of the Cat-B in cytosol stimulated by berberine, an isoquinoline alkaloid, were associated with caspase-independent cell death in colon tumor cells [38]. These results indicated that GEF plus SAL induced caspase-independent cell death by lysosomal cathepsins. Nevertheless, the molecular mechanisms underlying caspase-independent cell death exerted by cathepsins on tumor cells are still poorly understood.

**Figure 9:**
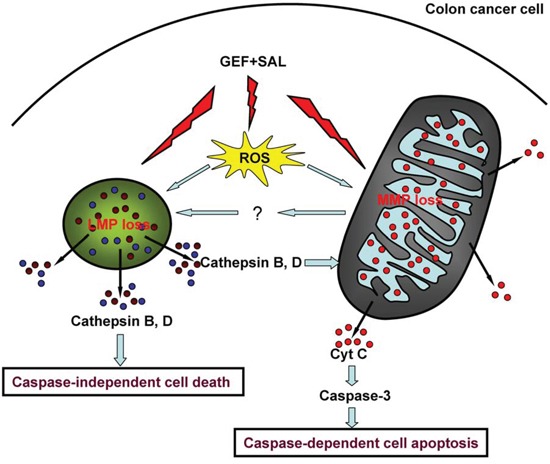
A model of cell death induced by gefitinib and salinomycin in combination Salinomycin (SAL) and gefitinib (GEF) acted together to enhance production of reactive oxygen species (ROS), loss of mitochondrial membrane potential (MMP) and lysosomal membrane potential (LMP). The ROS production induced by GEF and SAL contributed loss of LMP and MMP. SAL and GEF cooperated to trigger LMP and cytoplasmic release of cathepsin B and D. And then, cytoplasmic cathepsin B and D contributed to the loss of MMP and caspase-independent cell death. Moreover, cytoplasmic cathepsin B and D enhanced cyto-c (cytochrome c) translocation into the cytosol and induced subsequently caspase-3 dependent cell apoptosis. Additionally, the two drugs cooperated to trigger mitochondrial permeability, which contributed to the loss of LMP.

In addition, MPT pore sealing agents (carnitine and cyclosporine) repressed decline of lysosomal membrane potential induced by GEF plus SAL. Previous studies showed blocking MPT pore with cyclosporin A and bongkrekic acid prevents lysosomal rupture induced by DNA damage drugs in U937 cells [39]. However, little is known about the molecular mechanisms of lysosomal rupture induced by mitochondrial membrane permeabilization. All these data suggested SAL and GEF synergized to induce cells apoptosis via mitochondrial-lysosomal cross-talk in colorectal cancer cells (Figure 9).

Since SAL was currently under evaluation as single agent against breast cancer in early clinical trials, and GEF was already in clinical use for lung cancer and colorectal cancer. Thus it was feasible that such a combination treatment might in principle be transferred into clinical application. In conclusion, our studies provided novel insights into the synergistic interaction of SAL and GEF, and showed the combination of the two drugs could be a novel and promising therapeutic approach to the treatment of colorectal cancer, which warrants further investigation in a clinical setting.

## MATERIALS AND METHODS

### Reagents, cell culture and drugs treatment

Salinomycin (SAL), gefitinib (GEF), erlotinib (ERL), AEE-788 (AEE), afatinib (AFA) and dacomitinib (DAC) were purchased from Selleck Chemicals LLC (Houston TX, USA). N-acetyl-L-cysteine (NAC), vitamin C (Vit-C), dimethyl sulfoxide (DMSO), cyclosporine, carnitine, pepstatin A (Pep-A), CA-074-Me (CA), E64d, z-Val-Ala-Asp-fluoromethylketone (z-VAD-fmk), Z-Leu-Glu(OMe)-His-Asp(OMe)-FMK (Z-LEHD-FMK), Necrostatin 1 (Nec1) and Necrostatin 5 (Nec5) were purchased from Sigma (St. Louis, MO, USA). Vitamin C was dissolved in water. Other reagents were dissolved in dimethyl sulfoxide (DMSO) and stored at −80°C.

The human colon cancer-derived cell lines HCT-116, SW1116, SW480, HT-29, LOVO and NCM460 were obtained from the American Type Culture Collection (ATCC). The GEF-resistant SW1116 cells (SW1116-GEF) were established from a parental SW1116 cell line after continuously exposing SW1116 cells to low dose of GEF. SW1116-GEF cells were 8.2-fold more resistant than the parental SW1116 cells to GEF. All cell lines have a stringent quality control in cell authenticity and have incorporated short-tandem repeat (STR) profiling for cell line validation. All cells were cultured as previously described [40].

For drugs treatment, stock solutions were diluted to the indicated final concentrations with growth medium just before use. Prior to drugs treatment, cells were incubated for at least 8 h and thereafter replaced with fresh media containing drugs; control cells received an equivalent amount of DMSO.

### Cell viability and clonogenic cell survival

CCK8 assay was conducted to assess the cell viability. Briefly, cells were plated into 96-well plates at a density of 0.5–1 × 10^4^ cells per well and incubated for at least 8 h in a 5% CO_2_ atmosphere at 37°C before exposure to drugs. The media were then removed, and cells were treated with drugs. After the cells were incubated for indicated time, CCK8 reagent were added to each well and the plate was incubated for another 2 h at 37°C. Absorbance of the media was then measured using a Micro-plate Reader (Bio-Rad, Hercules, CA) at 450 nm. This assay was conducted in triplicate. Clonogenic cell survival experiments were performed as previously described [40].

### Combination index

For combination treatment of SAL and/or GEF, CCK8 assay data were converted to fraction of growth affected by the individual drug or the combination treated cells compared with untreated cells and analysed using CalcuSyn software (Biosoft, Ferguson, MO, USA) to determine whether the combination was synergistic. This program is based upon the Chou–Talalay equation [41], which calculates a combination index (CI). The general equation for the classic isobologram is given by: CI = (D)_1_/(Dx)_1_ + (D)_2_/(Dx)_2_. Where Dx indicates the dose of one compound alone required to produce an effect, (D)_1_ and (D)_2_ are the doses of compounds 1 and 2, respectively, necessary to produce the same effect in combination. From this analysis, the combined effects of the two compounds can be summarized as follows: CI < 1, CI = 1, CI > 1 indicate synergistic, additive and antagonistic effects, respectively [42].

### Plasmid transfection and RNA interference

Cells were transfected with pBabe puro-KRAS G12V (Addgene, 9052) and the corresponding empty vector pBabe puro as previously described [43].

siRNAs for down-regulating cathepsin B and D gene expression were done by transfection of RNA oligonucleotides with lipofectamine 2000 (Invitrogen, USA) according to the manufacturer's instructions as previously described [43]. The negative control (NC) siRNA and siRNAs against cathepsin B and D were synthesized by Shanghai GenePharma Co. For cathepsin B and D: 5′-TGAGCTGGTCAACTATGTC-3′ and 5′-GAACATCTTCTCCTTCTAC-3′, respectively.

### Cell cycle and apoptosis assays

Measurement of cell cycle was conducted by propidium iodide (PI) analysis. Briefly, single-cell suspensions were fixed in ice-cold 70% ethanol overnight, washed twice in PBS. And then cells were incubated with PI staining solution [0.1% Triton X-100 (Sigma-Aldrich), 50 μg/mL PI (Sigma-Aldrich), and 100 μg/ml DNase-free RNase A (Sigma-Aldrich) in PBS] for at least 30 min in dark at RT, and monitored with the FL3 channel in a FACS Calibur™ flow cytometer. The Sub G_1_ peak was utilized as a measure of apoptosis. The cell cycle data was analyzed with FlowJo V7 software (Tree Star, Oregon, US).

Cells apoptosis were measured by Annexin V-FITC (fluorescein isothiocyanate)/PI analysis as described previously [40].

### Measurement of lysosomal membrane potential (LMP) and mitochondrial membrane potential (MMP)

LMP and MMP were assessed by acridine orange (AO) and JC-1 staining respectively. To determine the assessment of LMP, cells were exposed to 1 mM of the lysosomotropic metachromatic fluorophore AO for 20 min at 37°C. And then cells were washed and resuspended in PBS. Changes in LMP were monitored with the FL3 channel in a FACS CaliburTM flow cytometer. For the assessment of MMP, cells were incubated with 10 μg/mL JC-1 in growth culture medium for 15 min at 37°C, washed twice, and resuspended in PBS and followed by determination of the JC-1 fluorescence with the FL1 and FL2 channels by FACS Calibur™ flow cytometry (BD Biosciences, San Jose, CA, USA).

### Western blot analysis

Total proteins were isolated from cells with RIPA lysis buffer. The protein concentration was determined by Bradford dye method. Equal amounts (20 to 50 μg) of cell extract were subjected to electrophoresis in 6–15% sodium dodecyl sulfate-polyacrylamide (SDS-PAGE) and transferred to PVDF or nitrocellulose membranes (Millipore, Darmstadt, Germany) for antibody blotting. The membranes were blocked and then incubated with Cathepsin B, Cathepsin D, COX IV, Cytochrome c, phospho-EGFR (Tyr1068), EGFR, phospho-AKT (Ser473) and Actin antibodies (all from Cell Signaling Technologies, Massachusetts, USA), γ-tubulin and Ras (all from Abcam), AKT (Santa Cruz) overnight at 4°C. Subsequently, the membranes were incubated with a HRP-conjugated anti-mouse or -rabbit secondary antibody (Protein Tech Group, Chicago, IL) at RT for 1 h. The protein bands were visualized using an enhanced chemiluminescence reagent (ECL) kit (GE Healthcare; Munich, Germany), according to the manufacturer's instructions.

### Measurement of intracellular reactive oxygen species (ROS) generation

ROS generation inside living cells was detected using flow cytometer with Dichloro-dihydro-fluorescein diacetate (DCFH-DA) (Wako Ltd, Osaka, Japan). Cells were loaded with 10 μM DCFH-DA for 30 min at 37°C in the dark. Subsequently cells were assayed with the FL1 channel by FACS Calibur™ flow cytometer as described in detail previously [44].

### Caspase activity assay and cytosolic cathepsin activity assay

Assays of caspase-9 and caspase-3 activity were carried out by using caspase-9 assay kit (abcam, ab65607) and caspase-3 assay kit (abcam, ab39383) according to the manufacturer's protocol as described previously [40].

Assays of cathepsin B (Cat-B) and cathepsin D (Cat-D) activity were carried out by using cathepsin B assay kit (abcam, ab65300) and cathepsin D assay kit (abcam, ab65302) according to the manufacturer's protocol. Briefly, 50 μg of cytosolic fraction protein prepared from control or stimulated cells was added up to the final reactive to 200 μL per well in a 96-well plate. Aliquots of assay volume were treated with 140 mM site-specific substrates in assay buffer at 37°C with 10 mM DTT for 0.5 h. Cleavage of the preferred Cat-B substrate [sequence RR labeled with AFC (amino-4-trifluoromethyl coumarin)] and Cat-D substrate [sequence GKPILFFRLK(Dnp)-D-R-NH2, labeled with MCA] by Cat-B and Cat-D release AFC and MCA respectively. The AFC fluorescence is measured at 400 nm excitation and 505 nm emission, and the MCA fluorescence is measured at 328 nm excitation 460 nm emission with a VersaFluor Fluorometer (Bio-Rad, Hercules, CA) respectively. The relative fluorescent units (RFU) were normalized with protein concentrations.

### Preparation of cytosolic and mitochondrial fractions

Cytoplasmic and mitochondrial fractions were prepared using a ProteoExtract-Subcellular Proteome Extraction Kit (Calbiochem) according to the manufacturer's instructions. The purity of each fraction was analyzed by Western blot using antibodies against γ–tubulin (cytoplasmic fraction) and COX IV (mitochondrial fraction).

### Xenografted colon cancer cells in nude mice

Athymic BALB/c nude mice (4–6 weeks old) were obtained from Si-Lai-Ke-Jing-Da Experimental Animal Co. Ltd (Changsha, China). All of the procedures of animal experiments were performed according to approved protocols and in accordance with the guidelines of the Guide for the Care and Use of Laboratory Animals (Institute of Laboratory Animal Resources, Commission on Life Sciences, National Research Council). It was approved by the Institutional Animal Care and Use Committee of our university (South China Normal University, Guangzhou, China). Mice were implanted subcutaneously into the right flanks with 2 × 10^6^ SW1116, SW1116-GEF and HCT-116 cells (6 mice in each group). After 10 days, the tumors became palpable. And then all mice were divided into four groups (6 mice per group) at random. For drugs treatment, 15 mg/kg or 40 mg/kg gefitinib, 30 mg/kg SAL, or combination of them dissolved in DMSO at a volume of 20 μL was administered to nude mice by intraperitoneal injection once per two days for 10 days. Tumor volume was measured two times a week by using calipers (as indicated at each time point) for 25 days. The tumor volume was estimated by the following formula: length×(width)×(width) × 3.14/6. The mice whole body weight was measured two times a week as indicated at each time point. All mice were euthanized by intraperitoneal injection of 200 mg/kg pentobarbital at the end of the experiment.

### Statistics

All experiments were repeated three times and were expressed as mean ± SD. *P* values were calculated using student's *t* test and *P* value <0.05 was considered significant. Statistical analysis was analyzed using the Statistical Package for Social Sciences (SPSS) software (version 16.0).

## SUPPLEMENTARY MATERIALS AND METHODS FIGURES


