# Supplementary material for: Synergistic induction of apoptosis by salinomycin and gefitinib through lysosomal and mitochondrial dependent pathway overcomes gefitinib resistance in colorectal cancer
Source: Oncotarget. 2015 Oct 7;8(14):22414–32. doi: 10.18632/oncotarget.5628 (PMC5410233; doi:10.18632/oncotarget.5628)
Supplement: Supplementary file 1 [file oncotarget-08-22414-s001.pdf]

SUPPLEMENTARY MATERIALS AND METHODS

CELL INVASION ASSAY

Cell invasion assay was performed in 24-well transwell chambers (Costar, Cambridge, MA, USA) containing polycarbonate filters with 8-μm pores coated with Matrigel™ (1 mg/mL, BD Sciences, San Jose, CA, USA). Briefly, 3×10^4 cells in 500 μL of serum-free medium were seeded into the upper chamber, and the lower chamber was filled with 800 μL of medium containing 10% FBS. After a 24-48 h incubation, the non-invasive cells were removed with a cotton swab, the number of cells that had invaded through the basement membrane were counted following staining with DAPI solution. Finally, the cell numbers were counted and averaged in six random fields at a magnification of 100 ×.

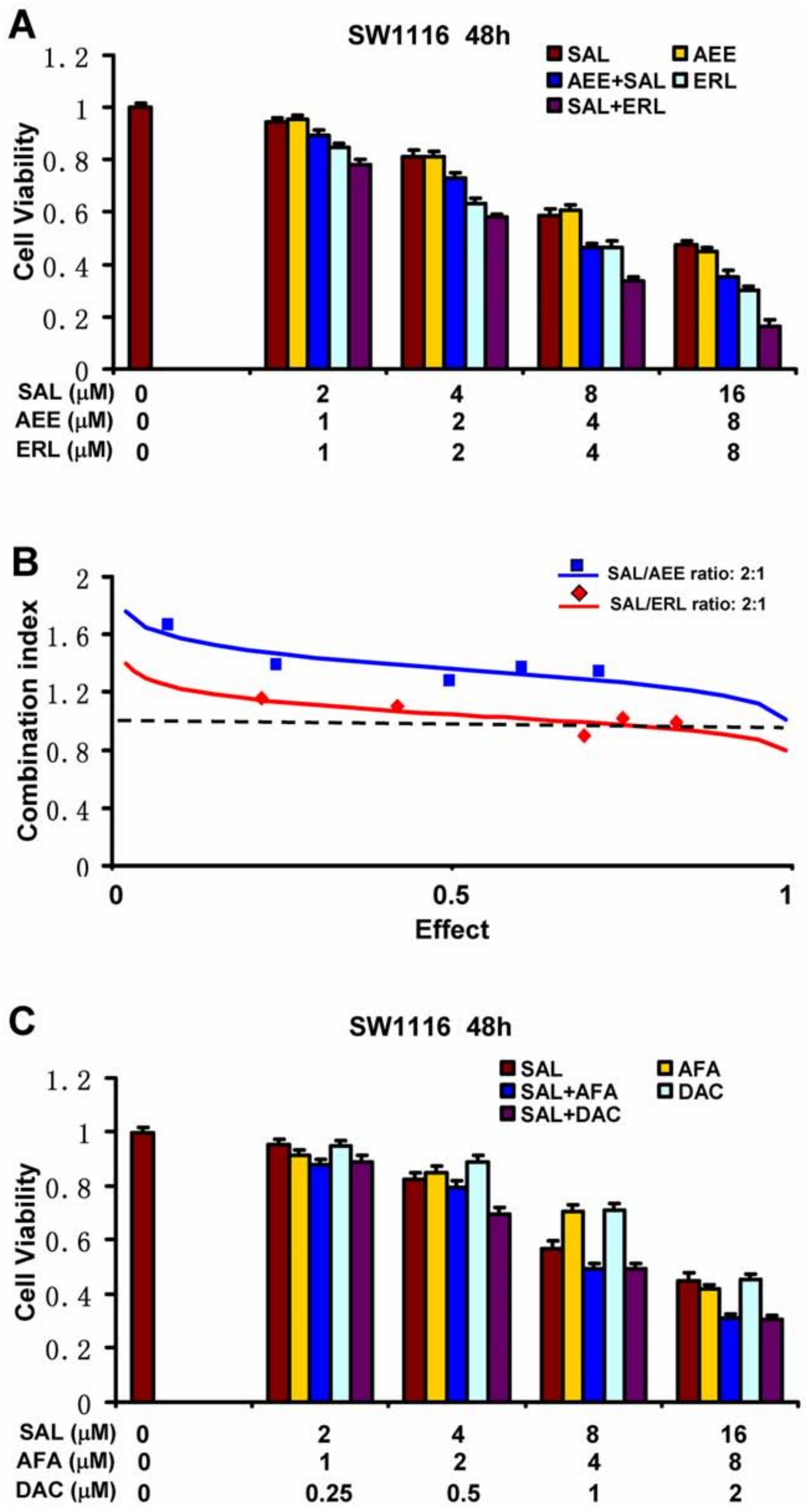

Supplementary Figure S1: Anti-proliferation effects of salinomycin (SAL) and erlotinib (ERL), AEE-788 (AEE), afatinib (AFA), dacomitinib (DAC) against SW1116 cells. A. and **C.** Cell viability was assessed by CCK8 assay. Mean ± S.D. of three independent experiments performed in triplicate are shown.

(*Continued*)

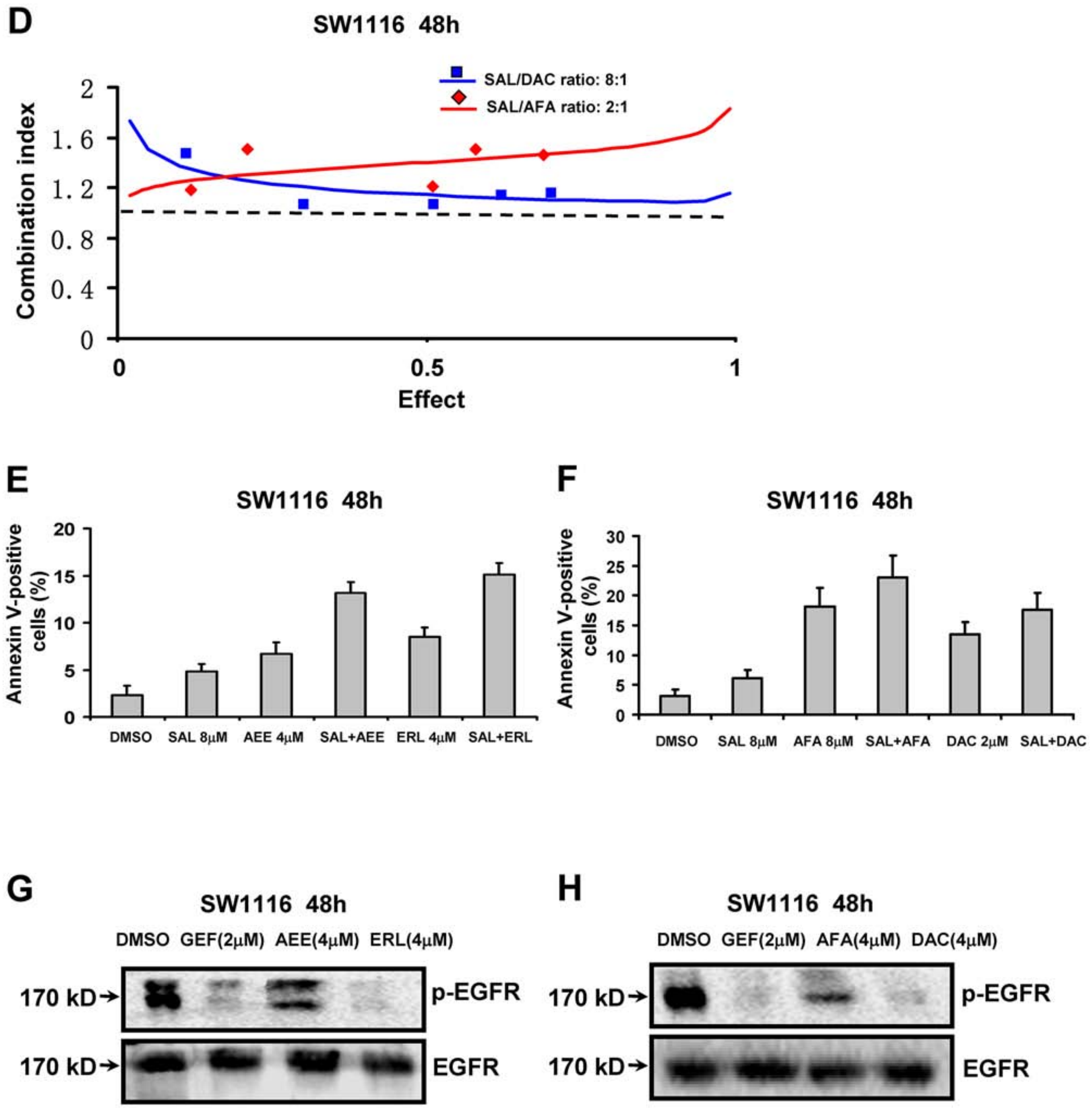

Supplementary Figure S1: (*Continued*) B and **D.** The combination index (CI) values were calculated according to the Chou–Talalay's method at the 48 h time point, with the biological response being expressed as the fraction of affected (Fa) cells. The data are representative of three independent experiments. **E** and **F.** SW1116 cells were treated with indicated concentrations of SAL and AEE, ERL, AFA or DAC alone, in combination for 48 h. Cell apoptosis was assessed by Annexin V-FITC/PI staining assay by flow cytometry. Columns, mean of three determinations; bars, S.D.. **G.** and **H.** SW1116 cells were treated for 48 h with the indicated concentrations of GEF, ERL, AEE, AFA and DAC. The p-EGFR and EGFR were detected by Western blot analysis. This experiment was repeated thrice.

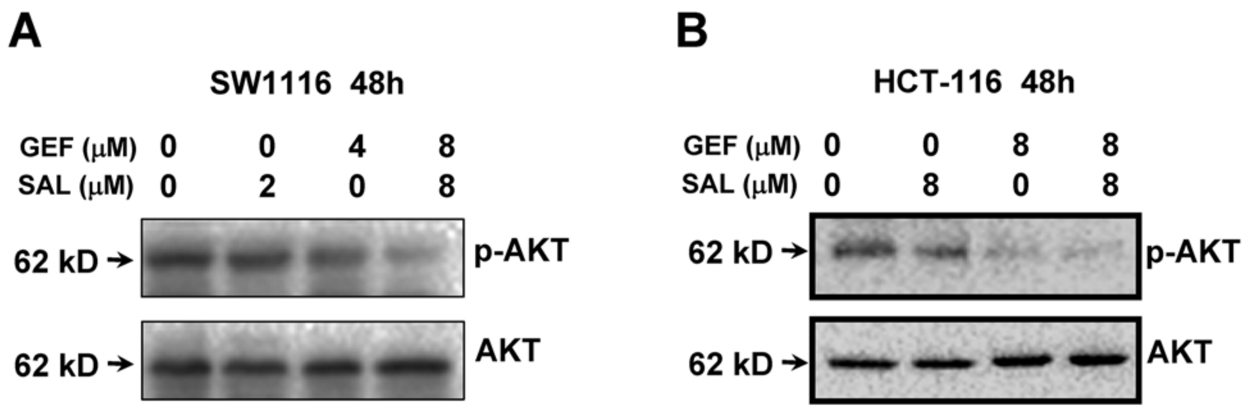

Supplementary Figure S2: The combination of salinomycin (SAL) and erlotinib (ERL) inhibits AKT activity. A. and **B.** SW1116 cells were treated for 48 h with the indicated concentrations of GEF and SAL. The p-AKT and AKT were detected by Western blot analysis. This experiment was repeated thrice.

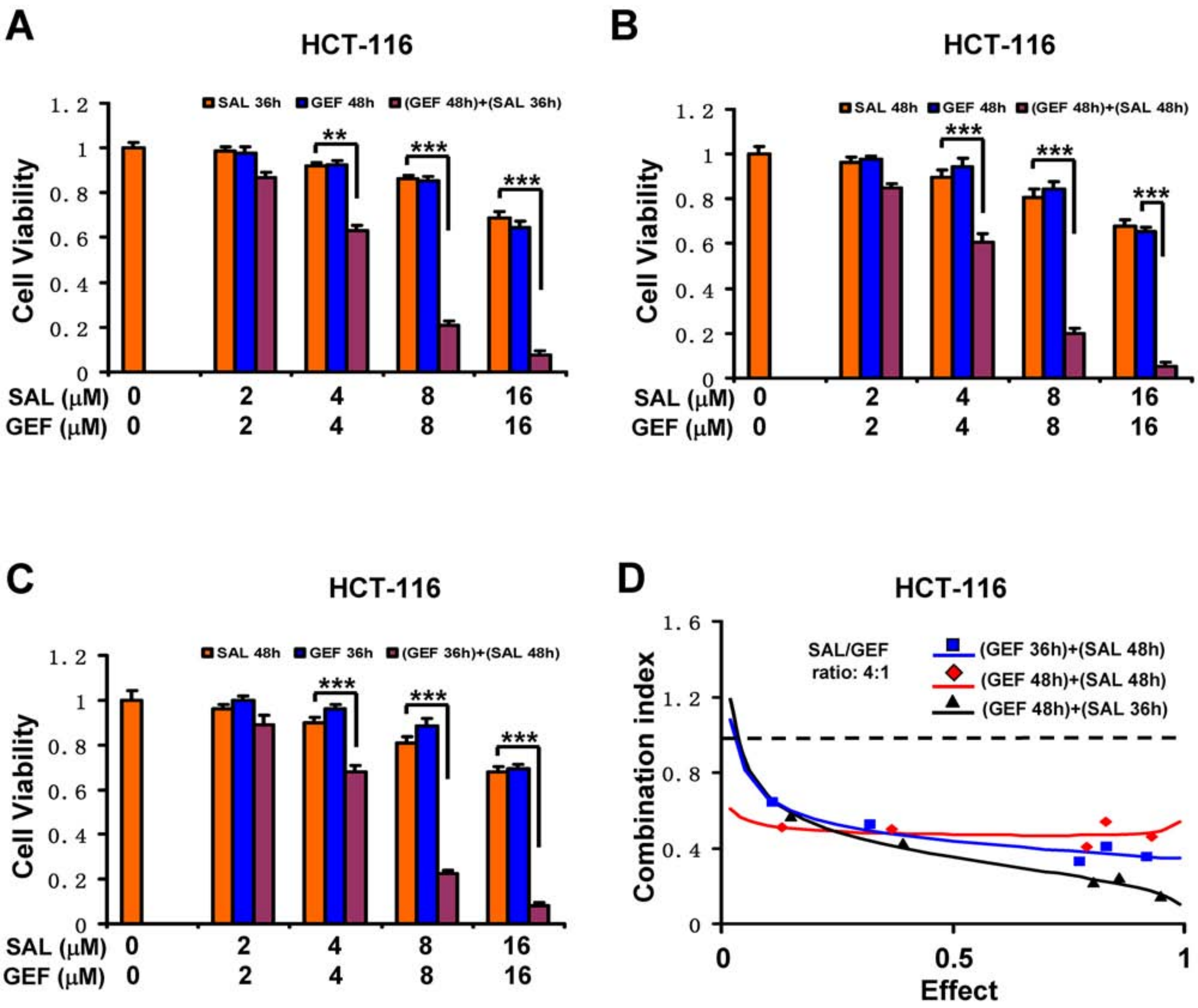

Supplementary Figure S3: Synergistic antineoplastic effects induced by gefitinib and salinomycin in colorectal cancer cells. A. HCT-116 cells were pretreated for 12 h with indicative dose of GEF and subsequentely with indicative dose of SAL for additional 36 h. **B.** HCT-116 cells were treated for 48 h with indicative dose of GEF and SAL. **C.** HCT-116 cells were pretreated for 12 h with indicative dose of SAL and subsequentely with indicative dose of GEF for additional 36 h. And then cells were assessed for viability by CCK8 assay. Mean ± S.D. of three independent experiments performed in triplicate are shown. $**P < 0.01$, $***P < 0.001$. **D.** HCT-116 cells were treated and processed as in A-D. The CI values for GEF and SAL were calculated according to the Chou-Talalay's method. Rectangle, diamond and triangle symbol designated the CI value for each Fa in SW1116 cells with three different sequences of GEF and SAL. The data are representative of three independent experiments.

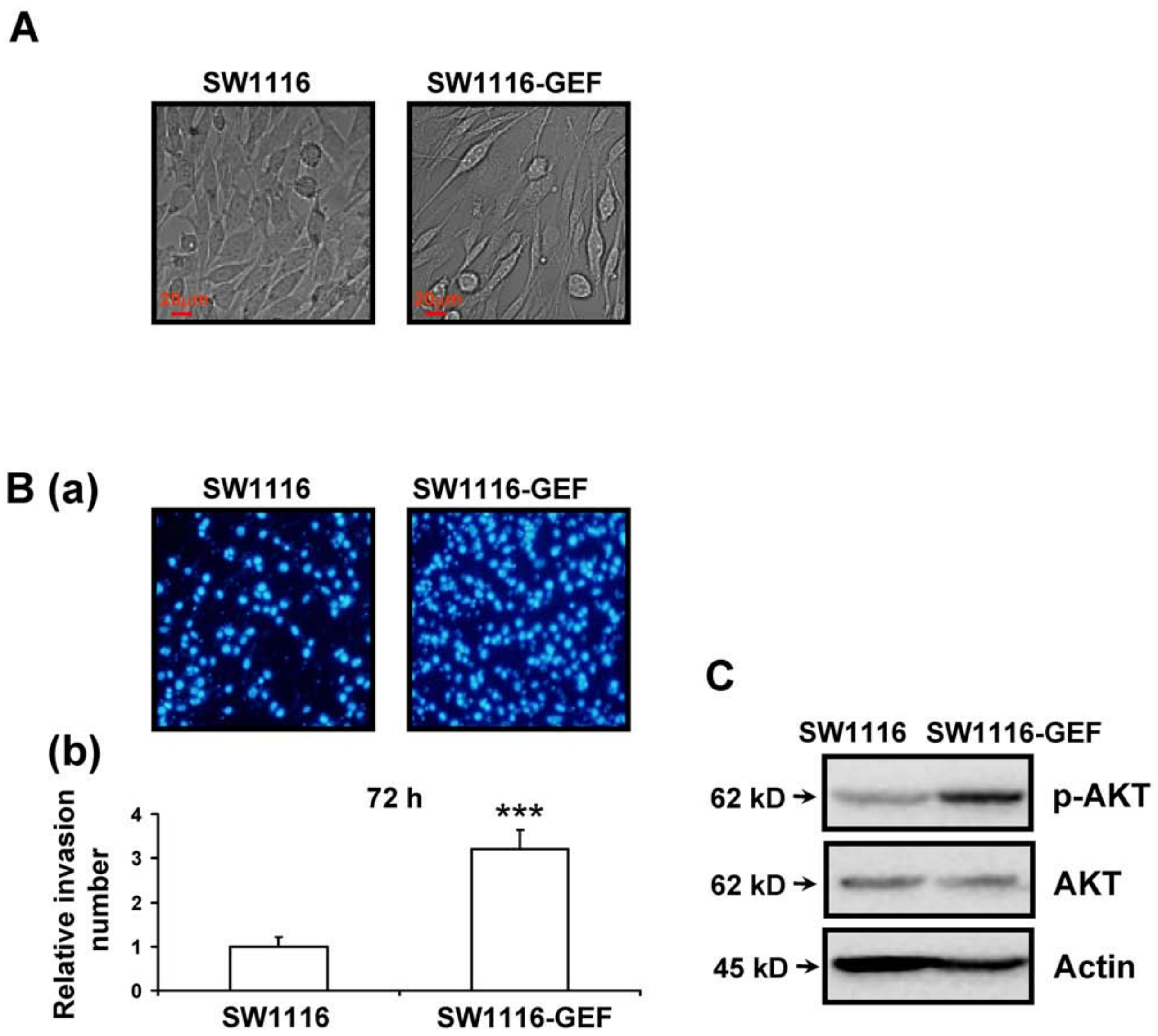

Supplementary Figure S4: Gefitinib-resistant colorectal cancer cells exerts higher invasive potency. A. Images were recorded using a digital camera with 1280 × 1280 pixels resolution. Magnification 400. Scale bar: 20 μm. **B.** Invasion rates of SW1116 and SW1116-GEF cells were quantified by counting the migrated cells in five random fields. (a) One representative of three independent experiments was shown, original magnification × 200. (b) Data summarized three independent experiments in identical condition. Mean ± S.D.. ***$P < 0.001$.

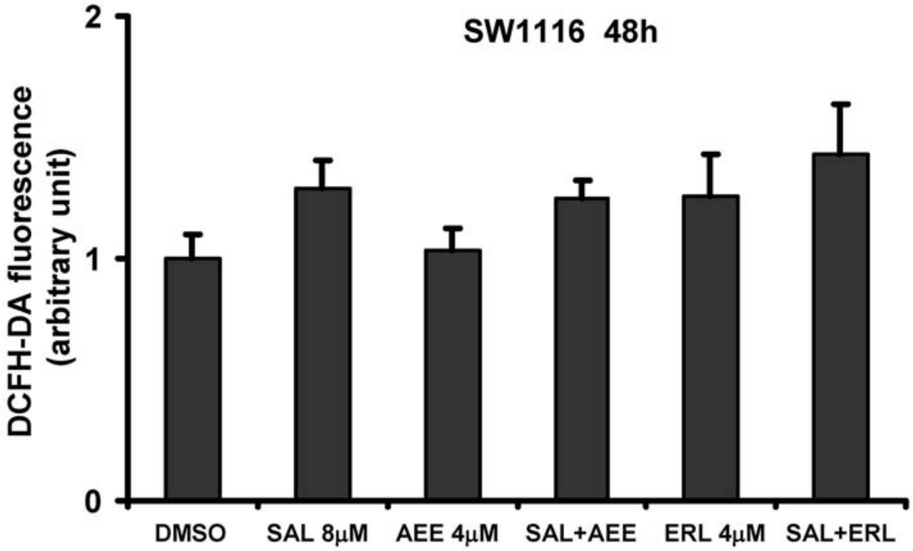

Supplementary Figure S5: The combination of salinomycin and erlotinib or AEE-788 fails to increase the ROS levels in SW1116 cells. The levels of ROS were measured with DCFH-DA staining by flow cytometric analyses. The levels of ROS were presented as fold change compared to the levels in control cells. Columns, mean of three determinations; bars, S.D.. Results shown were representative of three independent experiments.

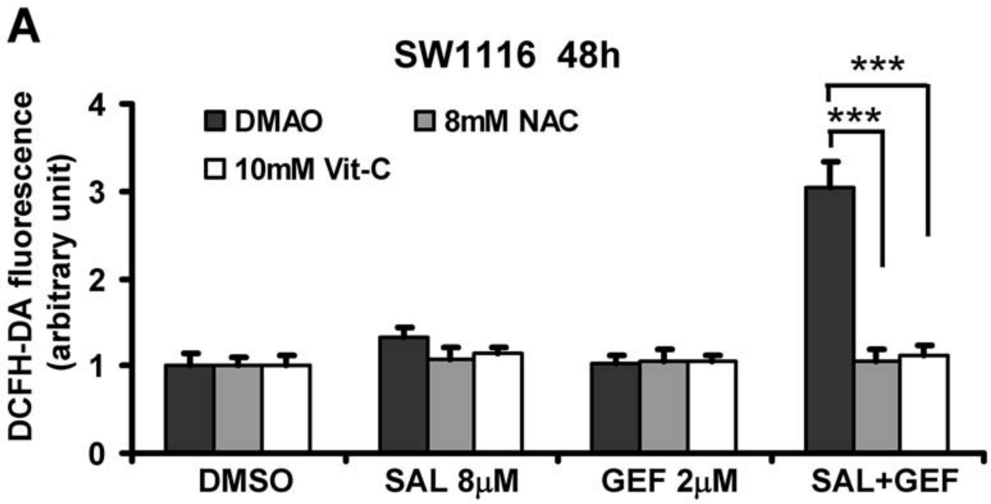

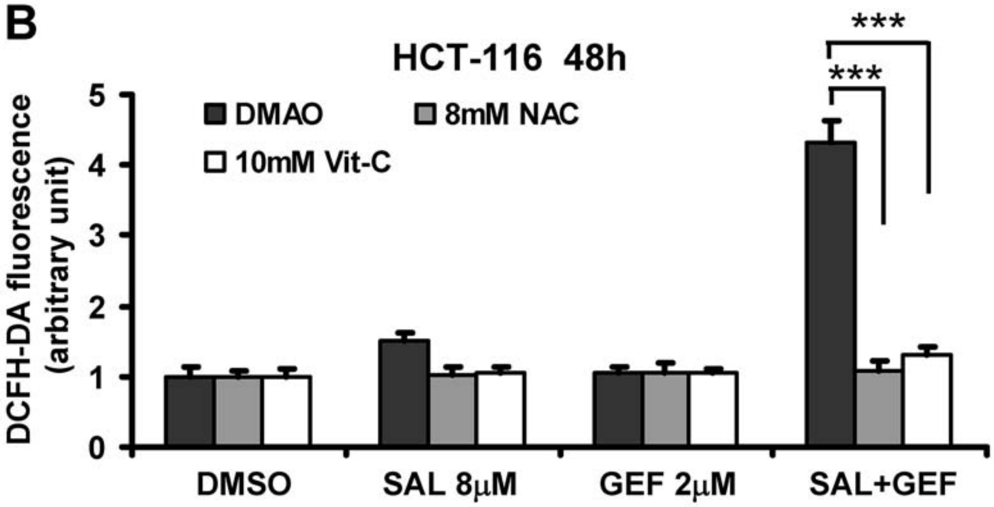

Supplementary Figure S6: The ROS production induced by gefitinib and salinomycin is completely abolished by N-acetylcysteine and vitamin C in colorectal cancer cells. A. and **B.** The levels of ROS were measured with DCFH-DA staining by flow cytometric analyses. The levels of ROS were presented as fold change compared to the levels in control cells. Columns, mean of three determinations; bars, S.D.. Results shown were representative of three independent experiments. ***$P < 0.001$, compared with DMSO-treated cells.

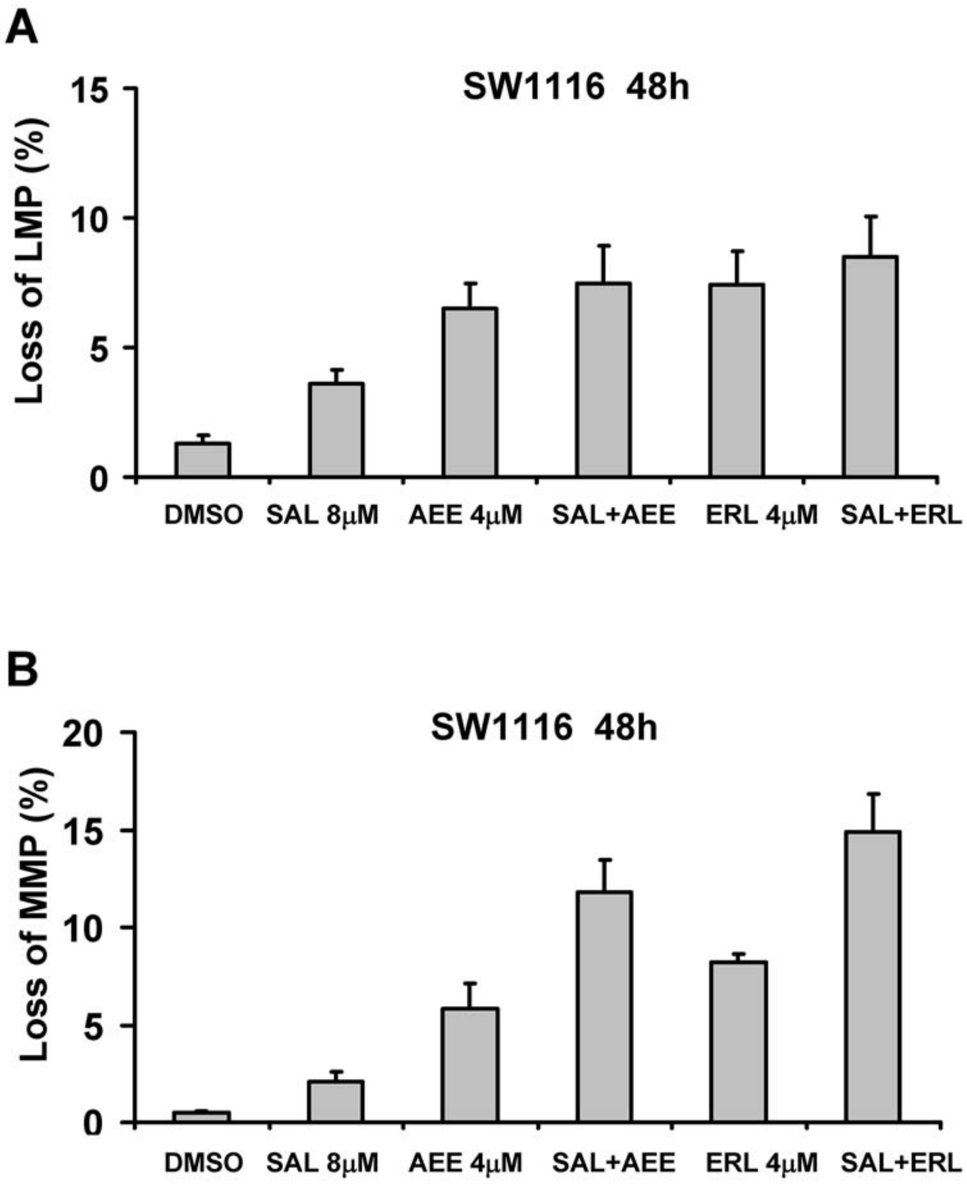

Supplementary Figure S7: The loss of lysosomal membrane potential and mitochondrial membrane potential induced by salinomycin and erlotinib, AEE-788 against SW1116 cells. A. The levels of lysosomal membrane potential (LMP) were measured with Acridine Orange (AO) staining by flow cytometric analyses. The percentage of cells with loss of LMP was shown. **B.** The levels of loss of mitochondrial membrane potential (MMP) were measured with JC-1 staining by flow cytometric analyses. The percentage of cells with loss of MMP was shown. This experiment was repeated thrice. Columns, mean; bars, S.D..

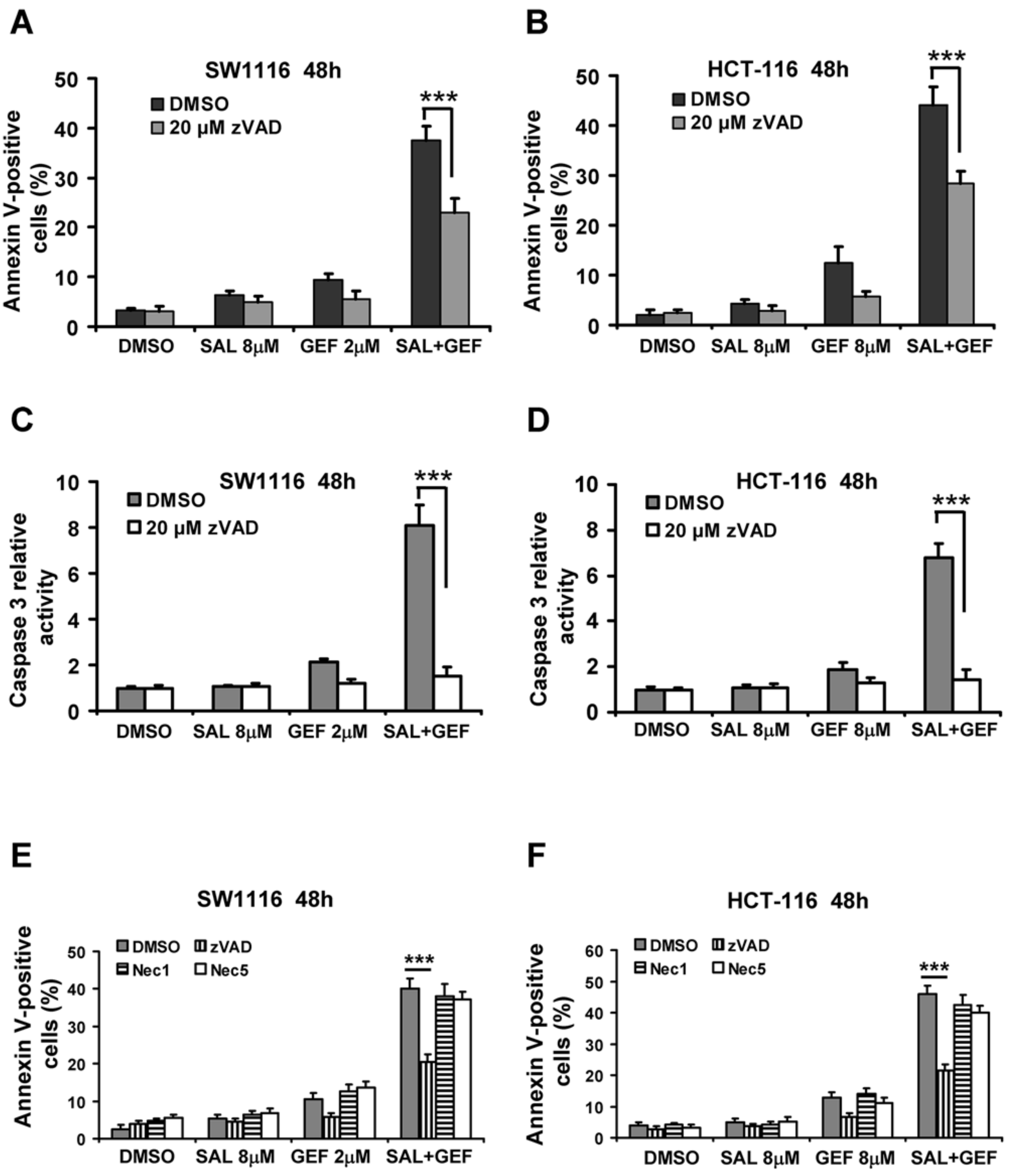

Supplementary Figure S8: Inhibition of caspase-3 attenuated colorectal cancer cells apoptosis induced by SAL plus GEF. (A-D) SW1116 and HCT-116 cells were pretreated with 20 μM z-VAD-fmk (zVAD) for 30 min before GEF and SAL treatment. **A.** and **B.** Cells were stained with Annexin V-FITC and propidium iodide (PI) and analyzed by flow cytometry. **C.** and **D.** The caspase activities were quantified as described under Methods. **E.** and **F.** SW1116 and HCT-116 cells were pretreated with 20 μM zVAD, 10 μM Necrostatin 1 (Nec1) and 10 μM Necrostatin 5 (Nec5) for 30 min before GEF and SAL treatment. Cells were stained with Annexin V-FITC and PI and analyzed by flow cytometry. Columns, mean of three determinations; bars, S.D.. Results shown were representative of three independent experiments. ***$P < 0.001$, compared with DMSO-treated cells.

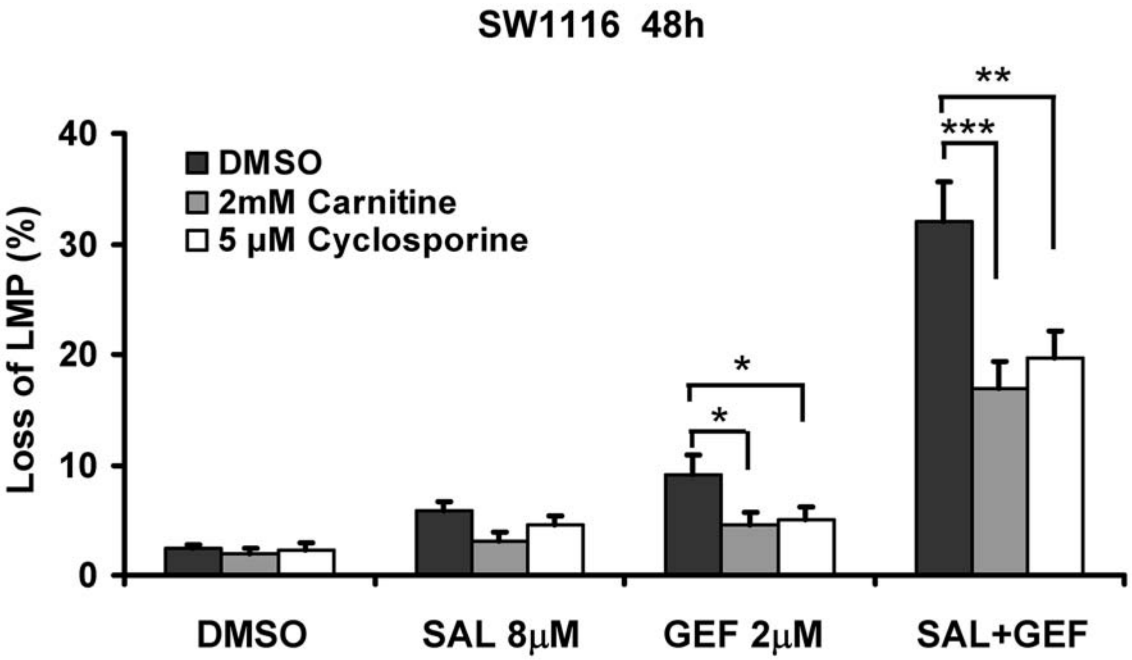

Supplementary Figure S9: Inhibition of mitochondrial permeability transition attenuated loss of LMP induced by SAL plus GEF. SW1116 cells were pretreated with 2mM carnitine and 5 μM cyclosporine for 30 min before GEF and SAL treatment. The levels of LMP were measured with Acridine Orange (AO) staining by flow cytometric analyses. The percentage of cells with loss of lysosomal membrane potential (LMP) was shown. Columns, mean of three determinations; bars, S.D.. Results shown were representative of three independent experiments. *$P < 0.05$, **$P < 0.01$, ***$P < 0.001$, compared with DMSO-treated cells.
